# Supplementary material for: Pyruvate Dehydrogenase Kinase 1 inhibition mediated oxidative phosphorylation enhancement in cartilage promotes osteoarthritis progression
Source: BMC Musculoskelet Disord. 2023 Jul 20;24:597. doi: 10.1186/s12891-023-06585-6 (PMC10357736; doi:10.1186/s12891-023-06585-6)
Supplement: Supplementary file 1 — Additional file 1. [file 12891_2023_6585_MOESM1_ESM.zip › BP.Gsea.1653623667859/GOBP_TOLERANCE_INDUCTION.html]

Details for gene set GOBP\_TOLERANCE\_INDUCTION[GSEA]

|  || Dataset | 55457\_collapsed\_to\_symbols.cls.cls #OA\_versus\_normal.cls.cls #OA\_versus\_normal\_repos |
| Phenotype | cls.cls#OA\_versus\_normal\_repos |
| Upregulated in class | normal |
| GeneSet | GOBP\_TOLERANCE\_INDUCTION |
| Enrichment Score (ES) | -0.54520434 |
| Normalized Enrichment Score (NES) | -1.840355 |
| Nominal p-value | 0.0017301039 |
| FDR q-value | 0.07462654 |
| FWER p-Value | 0.93 |
Table: GSEA Results Summary

  

Fig 1: Enrichment plot: GOBP\_TOLERANCE\_INDUCTION      
 Profile of the Running ES Score & Positions of GeneSet Members on the Rank Ordered List

  

| SYMBOL | TITLE | RANK IN GENE LIST | RANK METRIC SCORE | RUNNING ES | CORE ENRICHMENT || 1 | BLK | "BLK proto-oncogene, Src family tyrosine kinase [Source:HGNC Symbol;Acc:HGNC:1057]" | 2692 | 0.280 | -0.1804 | No |
| 2 | XKR8 | XK related 8 [Source:HGNC Symbol;Acc:HGNC:25508] | 2884 | 0.262 | -0.1684 | No |
| 3 | CD3E | CD3e molecule [Source:HGNC Symbol;Acc:HGNC:1674] | 2919 | 0.259 | -0.1445 | No |
| 4 | LILRB4 | leukocyte immunoglobulin like receptor B4 [Source:HGNC Symbol;Acc:HGNC:6608] | 3949 | 0.160 | -0.2080 | No |
| 5 | PSG9 | pregnancy specific beta-1-glycoprotein 9 [Source:HGNC Symbol;Acc:HGNC:9526] | 4254 | 0.132 | -0.2180 | No |
| 6 | IL2RA | interleukin 2 receptor subunit alpha [Source:HGNC Symbol;Acc:HGNC:6008] | 4292 | 0.129 | -0.2077 | No |
| 7 | LGALS9 | galectin 9 [Source:HGNC Symbol;Acc:HGNC:6570] | 4670 | 0.098 | -0.2269 | No |
| 8 | CCR4 | C-C motif chemokine receptor 4 [Source:HGNC Symbol;Acc:HGNC:1605] | 5108 | 0.063 | -0.2544 | No |
| 9 | MARCHF7 | membrane associated ring-CH-type finger 7 [Source:HGNC Symbol;Acc:HGNC:17393] | 6357 | -0.032 | -0.3480 | No |
| 10 | LYN | "LYN proto-oncogene, Src family tyrosine kinase [Source:HGNC Symbol;Acc:HGNC:6735]" | 6514 | -0.043 | -0.3557 | No |
| 11 | FOXP3 | forkhead box P3 [Source:HGNC Symbol;Acc:HGNC:6106] | 7035 | -0.082 | -0.3876 | No |
| 12 | PHLPP1 | PH domain and leucine rich repeat protein phosphatase 1 [Source:HGNC Symbol;Acc:HGNC:20610] | 7465 | -0.115 | -0.4091 | No |
| 13 | IDO1 | "indoleamine 2,3-dioxygenase 1 [Source:HGNC Symbol;Acc:HGNC:6059]" | 7782 | -0.140 | -0.4193 | No |
| 14 | TGFBR2 | transforming growth factor beta receptor 2 [Source:HGNC Symbol;Acc:HGNC:11773] | 8241 | -0.174 | -0.4370 | No |
| 15 | LILRB2 | leukocyte immunoglobulin like receptor B2 [Source:HGNC Symbol;Acc:HGNC:6606] | 9317 | -0.257 | -0.4942 | No |
| 16 | FOXJ1 | forkhead box J1 [Source:HGNC Symbol;Acc:HGNC:3816] | 9364 | -0.261 | -0.4710 | No |
| 17 | ICOS | inducible T cell costimulator [Source:HGNC Symbol;Acc:HGNC:5351] | 10320 | -0.353 | -0.5090 | Yes |
| 18 | PDCD1 | programmed cell death 1 [Source:HGNC Symbol;Acc:HGNC:8760] | 10584 | -0.382 | -0.4903 | Yes |
| 19 | HLA-G | "major histocompatibility complex, class I, G [Source:HGNC Symbol;Acc:HGNC:4964]" | 10869 | -0.413 | -0.4700 | Yes |
| 20 | CLC | Charcot-Leyden crystal galectin [Source:HGNC Symbol;Acc:HGNC:2014] | 11012 | -0.431 | -0.4368 | Yes |
| 21 | HLA-E | "major histocompatibility complex, class I, E [Source:HGNC Symbol;Acc:HGNC:4962]" | 11659 | -0.526 | -0.4330 | Yes |
| 22 | ITCH | itchy E3 ubiquitin protein ligase [Source:HGNC Symbol;Acc:HGNC:13890] | 11800 | -0.549 | -0.3876 | Yes |
| 23 | HMGB1 | high mobility group box 1 [Source:HGNC Symbol;Acc:HGNC:4983] | 12170 | -0.636 | -0.3510 | Yes |
| 24 | IRAK3 | interleukin 1 receptor associated kinase 3 [Source:HGNC Symbol;Acc:HGNC:17020] | 12383 | -0.703 | -0.2954 | Yes |
| 25 | HLA-B | "major histocompatibility complex, class I, B [Source:HGNC Symbol;Acc:HGNC:4932]" | 12534 | -0.764 | -0.2287 | Yes |
| 26 | AIRE | autoimmune regulator [Source:HGNC Symbol;Acc:HGNC:360] | 12610 | -0.804 | -0.1520 | Yes |
| 27 | TNFAIP3 | TNF alpha induced protein 3 [Source:HGNC Symbol;Acc:HGNC:11896] | 12631 | -0.814 | -0.0701 | Yes |
| 28 | CBLB | Cbl proto-oncogene B [Source:HGNC Symbol;Acc:HGNC:1542] | 12725 | -0.887 | 0.0136 | Yes |
Table: GSEA details [plain text format]

  

Fig 2: GOBP\_TOLERANCE\_INDUCTION      
 Blue-Pink O' Gram in the Space of the Analyzed GeneSet

  

Fig 3: GOBP\_TOLERANCE\_INDUCTION: Random ES distribution      
 Gene set null distribution of ES for **GOBP\_TOLERANCE\_INDUCTION**
